# How to provide and pay for long term care of an aging population is an international concern

**DOI:** 10.1186/2045-4015-2-4

**Published:** 2013-01-23

**Authors:** Marsha Gold

**Affiliations:** 1 Mathematica Policy Research, 1100 1st Street NE, 12th floor, Washington DC, 20003, USA

## Abstract

As populations age, most industrialized nations are seeking to review the structure for their long term care programs with the goal of allocating better limited public resources to meet expanding needs. In this Commentary, I examine critical questions that define the way individual nations provide for the long term care needs of their aging populations. As examined by Asiskovitch, Israel’s programs appear, in cross-national context, to have a broader reach and rely more heavily on community based services. In the future, the challenge Israel may face involves maintaining aspects of its programs that probably account for its popular support and stability while it identifies better the extent of potential gaps in care for those with greater needs and how best to meet them.

## 

The article by Asiskovitch in this issue of the IJHPR provides a valuable in depth review of Israel’s experience with long term care [1]. Israel’s program focuses on home based personal care services, employing a model that has been relatively stable since 1988. As the author describes, the program serves a relatively large and growing share of elderly beneficiaries with an emphasis on community based care. Despite a shift from two to three levels of benefits that vary by income and dependency, concerns remain about the equity of benefit distribution, particularly for those with greatest needs. Meanwhile in Israel, as in the rest of the world, societal changes and an aging population are creating growing strains on existing institutions and programs [2]. In Israel, these strains raise issues regarding appropriate benefit levels, how benefits should be financed, and how the quality of services should be guaranteed.^1^

In this article, I seek to put Israel’s experience in broader international context by posing some critical questions that apply to understanding long term care policies of any nation. I then build on them with the goal of placing Israel’s policies in cross-national context. For simplicity, I focus on the elderly though similar and interrelated issues exist across the age spectrum, particularly for disabled populations, raising issues about relative priorities across subgroups of the population and the extent to which specialized systems should serve the aged.

### Key policy questions

#### What are the nation’s obligations in meeting the long term care needs of its population and how, if at all, does that obligation vary by income or need?

Fundamental beliefs about the role of government are reflected in how nations’ structure programs to meet the long term care needs of their aging populations. In most developed nations, public spending on long term care exceeds that of the private sector [3]. However, such expenditures fail to reflect unpaid help by family members and friends providing informal care that may be the mainstay of care, particularly for the broader aged population even as socio-demographic trends weaken essential infrastructure to support such care. Programs that seek to pay cash benefits to the aged so they can more autonomously choose who will provide their care potentially blur the boundaries between the public and private sectors and the formal and informal sources of support [4-6].

Ultimately philosophy is translated into reality by eligibility policies, program design, and funding levels. Nations differ on whether eligibility is universal or limited by income or wealth, at what levels such income or wealth limits apply, and whether benefits are an entitlement or discretionary and thus depend on the level of financing or services authorized and made available. Both the scope of benefits provided and the priorities that define who receives them also shape the nature of government commitments. With resources limited, there may be trade-offs in equity between commitments that aim for more universal eligibility versus those that target subgroups with more extensive needs, however values of a society are reflected in the particular criteria employed.

#### How should long term care services be paid for and financed?

Public sector financing may be through programs of social insurance supported by dedicated taxes that raise revenue to support universal benefits, publicly underwritten insurance to eligible populations, grant supported programs, tax incentives, or direct service provision. Private spending comes from individuals, private insurance plans that may mediate between individual payments and services, or in kind (and typically uncounted) caregiver support by family, friends or community, including private charitable organizations and services. Nations can be categorized by the total amount of spending on long term care, the government (that is public sector) share of that spending, or the ways in which distribution varies for those at diverse income levels or needs. The argument for some external source of support for long term care rests at least in part on the uncertainties that surround ultimate need for such services.^2^ Nations also differ in how distinctively separate their long term services may be from the way they finance and provide acute care. How to better integrate and coordinate care that may be provided across acute and long term sectors is a growing issue as the population ages [7].

#### Where should long term care services be provided?

Historically, public spending programs in many nations have tended to direct a high proportion of their funds towards those treated in institutional settings of care, reflecting both higher costs for those at risk for needing care in such institutions and historical patterns that have favored care in such settings. In 2000, most nations spent the majority of their public long term care services on institutional care despite variations across nations.^3^ There now is growing interest in maintaining individuals at home or at least in the community to the extent feasible. However it is not clear that community services are necessarily less expensive, though that is the hope and community care tends to be preferred by beneficiaries [8].

#### How can growth in need for care with an aging population be accommodated?

This is a critical question that most nations now face as aging populations confront economies that are growing more slowly than in the post WWII era when the population was young and societal rebuilding was a priority. A reality of limited resources in the face of growing demand is leading many nations to reconsider both their obligations and the way they structure benefits. While there is hope that program consolidation, reorganization, and efficiency gains can stretch limited resources, ultimately trade-offs are likely to be required and the way those tradeoffs are made will shape future commitments and the way benefits are distributed both by income and need.

#### Israel in cross national context

While definitional inconsistencies complicate cross national comparisons, Asiskovitch’s analysis (Table 8) suggests that Israel provides publicly financed long term care services to a larger share of the elderly population than many nations, while expending a smaller share of the GNP on such public spending. From the data in the paper, it is not possible to assess fully potential inequities that may occur if these patterns reflect failure to allocate sufficient resources to those most in need.

As a non-Israeli, two aspects of the Israel system appear noteworthy. First, in contrast to many nations, Israel’s long term care system appears to encourage community based support, which is important at a time when many nations are striving to rebalance their own systems. Second, the breadth of reach behind Israel’s program is striking at a time of limited resources. Arguably such reach contributes to popularity which has helped maintain the stability of the program over time. Going forward therefore, the key challenge is to maintain such positive features while better identifying the extent of unmet needs and determining how best to build on the program to better meet them.

## Competing interests

The author declares that she has no competing interests.

## Authors’ information

Marsha Gold, Sc.D. is a senior fellow at Mathematica Policy Research in Washington DC, an independent nonpartisan organization focused on public relevant research and analysis. Dr. Gold is a member of the editorial boards of *Health Affairs* and *Health Services Research* and has published extensively on the organization and financing for health care in the United States. Commentary on “The Long Term Care Insurance Program In Israel: Solidity with the Elderly in a Changing Society (Sharon Asiskovitch).
